# Environmental Regulation of the Distribution and Ecology of *Bdellovibrio* and Like Organisms

**DOI:** 10.3389/fmicb.2020.545070

**Published:** 2020-10-29

**Authors:** Henry N. Williams, Huan Chen

**Affiliations:** ^1^School of the Environment, Florida Agricultural and Mechanical University, Tallahassee, FL, United States; ^2^National High Magnetic Field Laboratory, Florida State University, Tallahassee, FL, United States

**Keywords:** *Bdellovibrio* and like organisms, *Halobacteriovorax*, environmental factors, predatory bacteria, prey susceptibility, predator-prey interactions

## Abstract

The impact of key environmental factors, salinity, prey, and temperature, on the survival and ecology of *Bdellovibrio* and like bacteria (BALOs), including the freshwater/terrestrial, non-halotolerant group and the halophilic *Halobacteriovorax* strains, has been assessed based on a review of data in the literature. These topics have been studied by numerous investigators for nearly six decades now, and much valuable information has been amassed and reported. The collective data shows that salinity, prey, and temperature play a major role in, not only the growth and survival of BALOs, but also the structure and composition of BALO communities and the distribution of the predators. Salinity is a major determinant in the selection of BALO habitats, distribution, prey bacteria, and systematics. Halophilic BALOs require salt for cellular functions and are found only in saltwater habitats, and prey primarily on saltwater bacteria. To the contrary, freshwater/terrestrial BALOs are non-halotolerant and inhibited by salt concentrations greater than 0.5%, and are restricted to freshwater, soils, and other low salt environments. They prey preferentially on bacteria in the same habitats. The halophilic BALOs are further separated on the basis of their tolerance to various salt concentrations. Some strains are found in low salt environments and others in high salt regions. *In situ* studies have demonstrated that salinity gradients in estuarine systems govern the type of BALO communities that will persist within a specific gradient. Bacterial prey for BALOs functions more than just being a substrate for the predators and include the potential for different prey species to structure the BALO community at the phylotype level. The pattern of susceptibility or resistance of various bacteria species has been used almost universally to differentiate strains of new BALO isolates. However, the method suffers from a lack of uniformity among different laboratories. The use of molecular methods such as comparative analysis of the 16S rDNA gene and metagenomics have provided more specific approaches to distinguished between isolates. Differences in temperature growth range among different BALO groups and strains have been demonstrated in many laboratory experiments. The temperature optima and growth range for the saltwater BALOs is typically lower than that of the freshwater/terrestrial BALOs. The collective data shows not only that environmental factors have a great impact on BALO ecology, but also how the various factors affect BALO populations in nature.

## Introduction

*“Everything is everywhere, but the environment selects”* (Bass Becking 1934). This review explores the relevancy of this hypothesis to the group of predatory bacteria, *Bdellovibrio* and like organisms (BALOs). The environment may determine the success of an organism to inhabit a particular niche, its abundance, cellular and metabolic functions, interactions with other members of the community, animate and inanimate, and other ecological considerations. An overview of the major environmental factors that impact the occurrence, distribution, multiplication, and interactions of BALOs is examined and discussed in this review.

The BALOs are obligate, predatory bacteria that mortally attack, invade and lodge in the intraperiplasmic space of their prey bacteria. Following multiplication within the intact, killed prey cell, the predator lyses the prey’s cell membrane releasing progeny cells and intracellular organic matter into the environment. These processes give BALOs the potential to control susceptible bacterial populations, alter microbial community structure, and contribute to biogeochemical cycling. BALOs are environmental organisms ubiquitous in fresh and salt waters, soils, sewage, and plant systems, but are also found in animal bodies. However, their role in the environment, and the impact of environmental factors on their distribution, survival, behavior, and predatory activities have not been extensively reviewed. Aspects of the topics have been covered in book chapters (Williams and Piñeiro, 2007; Im et al., 2020; Jurkevitch and Mitchell, 2020) and in some reviews (Varon and Shilo, 1980) written before many of the more recent advances. There are more reviews (Starr and Seidler, 1971; Rendulic et al., 2004; Sockett and Lambert, 2004; Negus et al., 2017) detailing the physiological, genomics, and cell cycle events of the predators than those related to their ecology. Potential links between these cellular processes and environmental factors have not been suitably addressed and cannot be until there is a greater comprehension of the impact of the environment on BALOs. This prompted us to undertake this effort to present an overview of what is known on the topics and discuss what remains to be discovered to guide future investigations. Our immersion in the topical areas covered has confirmed to us that such a treatise is well overdue.

**FIGURE 1:**
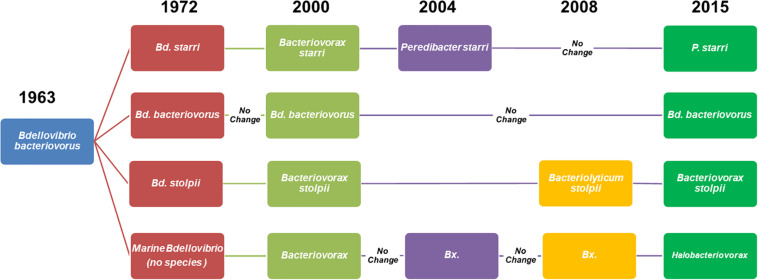
Schematic diagram of the evolution of the nomenclature and taxonomy of *Bdellovibrio*-like bacteria from their discovery (Stolp and Petzold, 1962) and naming (Stolp and Starr, 1963) as *Bdellovibrio bacteriovorus* to 2020. Subsequently, two major groups were defined by their requirement or tolerance to sodium chloride, the halophilic or marine *Bdellovibrio*, and the freshwater or terrestrial variety, *Bdellovibrio bacteriovorus*. In the early 1970s, *Bdellovibrio bacteriovorus* was split, establishing two new species, *Bdellovibrio. starrii* and *Bdellovibrio stolpii* (Seidler et al., 1972). Later, the original genus, *Bdellovibrio*, was split into two genera, *Bacteriovorax* (Baer et al., 2000) and *Peredibacter starrii* (Davidov and Jurkevitch, 2004). Then came the reclassification of *Bacteriovorax stolpii* as *Bacteriolyticum stolpii* (Piñeiro et al., 2008), leaving the genus *Bacteriovorax* comprised solely of the halophilic members. To correct an error, the original name *Bacteriovorax stolpii* was restored, as it is the type species for the genus *Bacteriovorax*. The previous saltwater “*Bacteriovorax*” was then assigned to a new genus, *Halobacteriovorax* (Koval et al., 2015).

We have focused on three highly relevant environmental factors, salinity, prey, and temperature, to BALO ecology. Although discussed separately, we are mindful that all, collectively and interactively, exert influence in the same moment in time and space on the behavior of these predatory bacteria. In the three decades following the discovery of BALOs in 1962, results from many well designed and executed experiments greatly advanced understanding of their ecology, and environmental factors that influenced their survival, interactions with prey bacteria, and distribution in nature. However, it was in the 1990s that the 16S rRNA gene as a phylogenetic tool to show the relatedness and distinctiveness among bacteria was applied to the BALOs (Donze et al., 1991). This event was of major significance, as BALOs are not readily grown in pure culture (the basis of physiological and phenotypic differentiation among bacteria) directly from environmental samples. This and other advances in molecular methods have led to great strides over the past 25 years in the advancement of knowledge on the ecology of BALOs as this review shows. Firstly, we believe it is helpful to clarify the nomenclature of BALOs as their systematics have been in a state of flux since shortly following their discovery. As readers refer to citations in this work, it will be found that the nomenclature of the predators in the literature has varied greatly, creating a conundrum. As of this review, there are four genera, three comprised of freshwater/terrestrial (F/T), non-halotolerant BALOs, *Bdellovibrio*, *Bacteriovorax*, and *Peredibacter*, and a single genus, *Halobacteriovorax*, for saltwater BALOs (Figure 1). The term BALOs includes the four genera and the epibiotic *Micavibrio*. The *Micavibrio* are not included in this review. To minimize confusion, in this article, we use the predator names as they appear in the original citation with their current names in parenthesis. This is found to be the case more so with the halophilic predators, which have been referred to by several genus names, whereas most studies of the freshwater/terrestrial predators have been on a single genus, *Bdellovibrio bacteriovorus*.

## Impact of Salinity

Following the original isolation of BALOs from soil, subsequent studies documented their presence in freshwater bodies (Shilo, 1966). Host-independent variants of these isolates could grow on media without added NaCl and were assumed to have little or no requirement for sodium ions (Seidler and Starr, 1969b). This was consistent with findings that the freshwater/terrestrial (F/T) BALOs were inhibited by salt concentrations greater than 0.5% (Varon and Shilo, 1968), which would classify them as non-halotolerant bacteria. There has been a report citing the detection of F/W BALOs in man-made saltwater systems using molecular techniques (Kandel et al., 2014). However, this was not confirmed by cultural methods. The first reports on the isolation of similar predatory bacteria from saltwater systems were by Shilo (1966) and Mitchell et al. (1967). The isolates were found to have the general features and lifecycle of the F/T, non-halotolerant *Bdellovibrio*, and became known as the “marine *Bdellovibrio*” (currently the *Halobacteriovorax*). Subsequently, host-independent marine *Bdellovibrio* variants were used to confirm that they have a specific requirement for sodium chloride for survival and growth (Reichelt and Baumann, 1974; Taylor et al., 1974). Thus, salt was recognized as a major environmental determinant in the ecology, physiology, genetics, and distribution of the *Halobacteriovorax*. They are found exclusively and ubiquitously in saltwater environments (Piñeiro et al., 2007), including diverse bodies as low saltwater estuaries, oceans, seas and high salinity saltern ponds (Sanchez-Amat and Torrella, 1989) and lakes (Piñeiro et al., 2004). The adaptation of the *Halobacteriovorax* to salt and other characters establishes them as a distinct genus apart from the non-halotolerant BALOs of the genera, *Bdellovibrio*, *Bacteriovorax*, and *Peredibacter.*

Following the first reports of *Bdellovibrio*-like bacteria in salt-water bodies (Shilo, 1966; Mitchell et al., 1967), investigations were undertaken to more fully describe their characteristics (Taylor et al., 1974; Miyamoto and Kuroda, 1975; Marbach et al., 1976). The first comprehensive study was reported by Taylor et al. (1974). Of 13 isolates from coastal waters off Oahu, HI (United States), all required sodium chloride for growth (plaque formation) on a semi-solid agar medium with various prey bacteria. The required sodium could not be replaced with KCl. The 13 isolates were divided into two groups, those that required at least 75 mM, and those requiring 100 mM (8 of the 13 isolates). Based on the time of plaque appearance and size, the optimum sodium ion concentration for the two groups was 125 and 150 mM, respectively. This is the first report of the division of the marine bdellovibrios (*Halobacteriovorax*) into subgroups based on their salinity growth range. Subsequent environmental studies confirmed such differentiation among the predators as observed in their distribution along salinity gradients in the Chesapeake Bay estuary (Piñeiro et al., 2013). Taylor et al. (1974) seminal investigation established previously unknown properties of the predators and protocols for future studies, including best type media and prey bacteria (see following section 3, “Impact of Prey”), which are still used. The requirement of *Halobacteriovorax* for salt reported by Reichelt and Baumann (1974) and Taylor et al. (1974) is consistent with that of other true marine bacteria.

Investigations by others confirmed the halophilic BALOs requirement for sodium chloride and revealed more information on their salinity growth ranges and dependency on other salts. A detailed report by Marbach et al. (1976) described the salinity requirements for marine *Bdellovibrio* (*Halobacteriovorax*) isolates from the Mediterranean Sea. Among ten isolates, the salinity growth range extended from 1.9 to 5.9%, with most between 2.38 and 4.75%. The requirement for NaCl could not be replaced by KCl as reported previously by Taylor et al. (1974). Growth of the *Halobacteriovorax* isolates was not detected in media without added KCl, MgCl_2_, or CaCl_2_. The requirement for these cations was confirmed by Marbach et al. (1976) and Bell and Latham (1975). This firmly established the halophilic predator’s requirements for cations other than sodium and further distinguished them from the F/T BALOs.

The Mediterranean and Pacific *Halobacteriovorax* isolates varied in their NaCl requirements. The Mediterranean strains grew between 100 and 400 mM NaCl (Marbach et al., 1976), whereas the minimum requirements for the Pacific Ocean isolates were below 100 mM (Taylor et al., 1974). Marbach and Shilo (1978) also showed variations among *Halobacteriovorax* strains in concentrations of cations required for growth. BM4 strain required five-fold greater concentrations of KCl and CaCl and 100-fold more MgCl_2_ than BM11. This is further evidence that *Halobacteriovorax* isolates are a diverse group in regards to salt requirements. This diversity is also seen in the genetic make-up of the predators as manifested in distinct 16S rDNA gene phylotypes that are associated with various salinity gradients (Piñeiro et al., 2013). Sodium may be a major factor in the greater genetic diversity of saltwater isolates than observed in F/T isolates.

To better understand the underlying mechanisms fostering the dependency of *Halobacteriovorax* on the various cations, Marbach and Shilo (1978) investigated the functions of the various cations at the cellular level. The investigators deciphered the role of each of the cations in specific cellular functions in the developmental stages of the life cycle of the *Halobacteriovorax*. In experimental trials, one of the four cations (Na^+^, K^+^, Mg^2+^, and Ca^2+^) was excluded from the growth medium in which the predator and prey were co-cultured. The effects of the deficient cation medium on the structure and function of the predatory cells were observed by light microscopy and compared to that in the complete medium with all four cations. In the absence of K^+^, predator motility was severely impaired with an average velocity of approximately 21 μm per sec, whereas, in the complete medium with K^+^, it was four-fold higher. BALOs are among the fastest bacteria. Their rapid motility is a critical function in tracking and attacking their prey to initiate the predators’ infection cycle including intraperiplasmic growth and multiplication. Any impairment in motility will decrease their predation efficiency and survival.

A Ca^2+^ deficiency was observed to inhibit the continuous growth cycles of the predators on the prey. The daughter cells released at the end of the first multiplication cycle were incapable of initiating a new infection cycle. The addition of CaCl_2_ to the deficient medium restored the predator’s attachment to its prey and infection cycle of intraperiplasmic growth. Mg^2+^ deficiency resulted in a prolonged and altered prey infection cycle. These and other observations by the investigators suggested that magnesium promotes attachment, penetration, bdelloplast formation and stabilization of the bdelloplast wall. This is consistent with the requirement of many marine bacteria for Mg^2+^ for structural integrity.

The collective results from these studies show that the cation effects on the *Halobacteriovorax* are specific and interdependent for certain functions in their infection and replication cycles. Without all four major cations, *Halobacteriovorax* predation would not occur and the predators as we know them would not exist. This is in contrast with the more general effects of cations on the infection cycles of the non-halotolerant BALOs. Due to their requirement for cations, *Halobacteriovorax* are considered true marine bacteria as defined by MacLeod (1965) and refutes the argument that their origin was sewage (Bell and Latham, 1975) or terrestrial habitats.

Investigations on the *Halobacteriovorax* through the mid-1980s were of predator isolates from low to medium salt (<5% salt) environs, oceans, seas, and estuaries. In the first comprehensive investigation of isolates from extreme saltwater habitats, Sanchez-Amat and Torrella (1989) reported consistent recovery of “halophilic bdellovibrios” (*Halobacteriovorax*) from high salinity (42 to 200 g/L total salts) solar evaporated ponds. The properties of these isolates were compared with halophilic predators similarly isolated from adjacent Mediterranean seawater samples. Among 13 isolates, four from seawater and nine from high salinity solar ponds, the salinity growth range extended from 2 to 12% (total salts) with the exception of three seawater isolates that grew at 1% salinity and one that did not grow above 6.5%. The optimum growth of all occurred between 3 and 5%. No isolate grew at 15% salinity, although a predator strain was recovered from a pond with 15.5% salinity. This prompted the investigators to test the salinity growth range of all isolates in broth culture instead of on agar plates. All isolates from the salt ponds grew at 15% salinity, but none of the seawater isolates, thus further distinguishing the predator isolates from seawater and high salt ponds. The predators were unable to prey in broth medium at 15% NaCl salinity, whereas predation was observed in the same medium “with a 15% total salts salinity.” This is the first report of *Halobacteriovorax* isolates as extreme halophiles. Prior to this study, the highest salinity growth range reported for halophilic predators was 5.9% (Marbach et al., 1976). The results show greater diversity of the predators in salt tolerance and greatly extend the range of salt environments in which predation by *Halobacteriovorax* occur.

Another extreme salinity habitat at even higher salinities in which salt water *Bdellovibrionaceae* (*Halobacteriovorax*) were recovered was The Great Salt Lake (Utah, United States). Piñeiro et al. (2004) isolated the predators from the lake where the typical salinity may range between 40 and 170 ppt. The isolates were phylogenetically related to strains from oceans and seas. However, they were able to prey more efficiently on Great Salt Lake bacteria than those from other saltwater bodies, which is evidence of their adaptation to the extreme salt environment.

**FIGURE 2:**
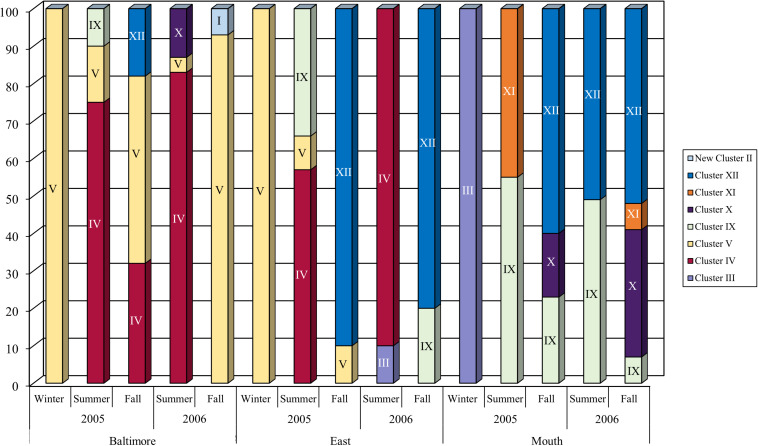
The succession of *Bacteriovorax* (now *Halobacteriovorax*) phylotypes isolated from the top and bottom water samples and sediment samples from three stations, Baltimore (oligohaline zone), East Middle (mesohaline zone), and Mouth (polyhaline zone) by season along Chesapeake Bay salinity gradient in 2005 and 2006. The percentages shown along the *Y*-axis are based on mean counts of *Halobacteriovorax* plaques from five transects of the Bay conducted in different seasons in a 2-year period over a salinity range between 5 and 31 ppt. Reprint from Piñeiro et al. (2013).

Further evidence of the influence of varying salt concentrations on the distribution of *Bacteriovorax* (*Halobacteriovorax*) is shown in results of a study in the Chesapeake Bay (United States), a large estuary with three salinity zones, oligohaline (0.5–5.0 ppt), mesohaline (5.0–18.0 ppt), and polyhaline (18.0 to 30.0 ppt) (Piñeiro et al., 2007; Montagna et al., 2012). The results show a highly diverse population of seven different *Halobacteriovorax* phylotypes (based on 97% or greater similarity of the 16S rRNA gene) along a transect of the Bay with distinct distribution patterns within the three salinity zones (Figure 2). Phylotype clusters IV and V were only found in the mesohaline zone, and Cluster XI only in the polyhaline zone. The mid-bay, representing the mesohaline zone, harbored some phylotypes from both the lower salinity regions of the mesohaline and the polyhaline zones. A significant difference was found between clusters occurring at salinities above and below 10 ppt. In Delaware Bay, a similar distribution pattern of phylotypes along the salinity gradient was observed (Richards et al., 2013). The only inconsistency with the Chesapeake Bay findings was that neither Cluster IV nor V was recovered from the Delaware Bay low salinity site 4. A possible explanation is the persistent, moderately high salinity at the site for prolonged periods, exceeding 20 ppt for five consecutive months. This is not conducive conditions for the low salinity predators (Clusters IV and V) to become established. Likewise, the lowest salinity at the site, 4.8 ppt, may have been too low to support the persistence of these two clusters.

Other studies have confirmed similar distribution patterns. A study of *Halobacteriovorax* in shrimp ponds and coastal waters in South China by Wen et al. (2009) did not address the association of phylotypes with various salinities. However, our analysis of the data revealed the same phylotype distribution pattern based on salinity, as observed in Chesapeake Bay (Piñeiro et al., 2013), although a different prey (*V. alginolyticus* as opposed to *V. parahaemolyticus*) was used, and the water bodies were vastly dissimilar. As in the Chesapeake Bay, phylotype XI was recovered from mesohaline to polyhaline waters, but mostly the mesohaline; phylotype X in polyhaline moderate salinity waters (16.94 to 30.75 ppt); and, phylotype IV in the mesohaline (low salinity waters, 8.26 to 18.44 ppt). These results confirm the selection of *Halobacteriovorax* phylotypes by salinity, and cluster IV as a low to moderate salinity strain.

Also, salinity has been shown to impact the association of *Halobacteriovorax* with surfaces in brackish waters. Kelley et al. (1997) observed that the association of the predators with submerged surfaces was significantly increased at salinities greater than 11‰ compared to those in lower-salinity waters. In freshwater (Gunpowder River, MD) with no measurable salinity, *Halobacteriovorax* did not colonize surfaces, but F/T BALOs did. The association of both *Halobacteriovorax* and F/T BALOs with surfaces has been reported by Kandel et al. (2014).

Of the known micropredators, the least studied are the BALOs. Among the BALOs, the halophilic *Halobacteriovorax* is understudied. The impact of salinity on the mode of life, and the very existence of *Halobacteriovorax* is remarkable, and perhaps previously under appreciated. In summary, the predation and intraperiplasmic growth cycle is dependent on cationic salts; the distribution of the halophilic predators is determined by salt; the systematics of BALOs is based in part on salt requirements; the susceptibility of bacteria to *Halobacteriovorax* may be influenced by salt, as halophilic bacteria are generally more susceptible to saltwater predators; salinity establishes specific environmental niches for distinct *Halobacteriovorax* phylotypes; and, salinity influences the association of the predators with surfaces. The finding of specific clusters (phylotypes IV and V) that only occurred in lower salinity brackish waters suggests that the low salt environment selected for distinct low salinity or estuarine strains of *Halobacteriovorax.* We have little doubt that salts have even a greater role on *Halobacteriovorax* than reported here.

## Impact of Prey

*Bdellovibrio* and like organisms (BALOs) are primarily obligate predatory bacteria. As such, they are dependent on nutrition, reproduction, and protection on prey bacteria. BALOs attack, invade, feed upon, grow, and ultimately multiply before lysing the prey and releasing daughter cells to repeat the cycle. Stable bdelloplasts have also been reported to extend the viability of the intracellular predators under certain starvation conditions (Sanchez-Amat and Torrella, 1990). Hence, prey is a critical environmental factor for the growth and survival of BALOs. From an ecological perspective, critical questions about prey include the following: Which bacteria are prey for BALOs? Where are the prey found? What factors determine their interaction with the BALOs? Do BALOs show preferences for which bacteria species they prey upon? Is there a difference in the bacteria species that are susceptible to halophilic versus non-halotolerant BALOs? What abundance of prey is required to support BALO populations? In this section, we review the literature addressing these questions.

### The First Prey and Prey Susceptibility Test

The first isolation of small, highly motile, predatory bacteria (later named *Bdellovibrio bacteriovorus*), by Stolp and Petzold in 1962 was serendipitous. The investigators were attempting to isolate from soil samples a bacteriophage against a plant pathogen, *Pseudomonas phaseolicola*, on which *Bdellovibrio* grew. Thus, this *Pseudomonas* species became the first known *Bdellovibrio* prey (Stolp and Petzold, 1962). Following this discovery, many efforts to isolate *Bdellovibrio* from soil or freshwater sources were reported. A common method used to characterize and distinguish new *Bdellovibrio* isolates from others was the prey susceptibility test. Differences in the patterns of susceptibility among the tested bacteria were used to differentiate multiple *Bdellovibrio* isolates (Supplementary Table 1) (Stolp and Starr, 1963). Prey-susceptibility tests also identified bacteria that were susceptible to BALOs. This information could be useful to determine which bacteria to use to recover BALOs and to provide a rough estimate of the number of prey bacteria available in the environment to the predators. Most studies report that BALOs prey only on Gram-negative bacteria, although some investigators have reported predation on Gram-positive bacteria (Qian, 1994; Najnine et al., 2020). Recent investigations show the predators obtain nutrients from some Gram-positive bacteria without penetrating the cell wall (Iebba et al., 2014; Waso et al., 2019). This important finding could expand sources of nutrients for the obligate predatory BALOs and deserves further study.

#### Halobacteriovorax and Prey

The isolation of *Bdellovibrio*-like bacteria from the marine environment was first reported by Shilo (1966). Described as “obligately halophilic,” the isolates were recovered from the Mediterranean Sea on *Ps. putida* and *Escherichia coli.* A year later, Mitchell et al. (1967) described a similar bacterium that preyed upon *E*. *coli* cells. The discovery of halophilic *Bdellovibrio* (*Halobacteriovorax*) in salt-water systems unveiled a new and different ecosystem inhabited by the predators.

The selection of *E. coli* as prey to isolate the first marine bdellovibrios (*Halobacteriovorax*) (Shilo, 1966; Mitchell et al., 1967) was a fortunate happenstance, as they generally prey more efficiently on native salt-water bacteria than on freshwater/soil bacteria (Taylor et al., 1974). We will address the question of which bacteria in saltwater and freshwater systems that autochthonous predators prey upon.

The first comprehensive investigation of bacteria susceptible to marine *Bdellovibrio* (*Halobacteriovorax*) was reported by Taylor et al. (1974). The results showed *V*. *parahaemolyticus* and other vibrios to be more susceptible than non-vibrio bacteria tested, and a preferred prey for *Halobacteriovorax*. Forty-two bacteria species, including marine and non-marine bacteria, were tested on a minimal and an enriched medium for their susceptibility to 13 *Halobacteriovorax* isolates from coastal waters around Oahu, HI (United States) (Supplementary Table 2). The marine bacteria, primarily *Beneckea*, now classified as *Vibrio* species (Baumann et al., 1980), were most susceptible (95.7% exclusive of one outlier, *B. nigrapulchrituda*, which was not susceptible to any *Halobacteriovorax* isolates). The *Photobacteria* and related bacteria were also highly susceptible (80%). On the other hand, the non-fermentative species (primarily species of *Alteromonas, Pseudomonas, Alcaligenes* and related bacteria) in both the marine and non-marine groups (*Pseudomonas* and *Acinetobacter*) were only 34.6% to 38.4% susceptible to *Halobacteriovorax* predation. The non-marine, eubacteria group included enteric species *E. coli*, *Salmonella typhimurium*, and *Aerobacter aerogenes*, were 53.8% susceptible. *V. cholera* and *Aeromonas formicans* were 76.9 and 100% susceptible, respectively. The non-fermentative *Acinetobacter* and *Pseudomonas* species were only 34.6% susceptible. A few other observations in this study are noteworthy. The *Halobacteriovorax* preyed with higher frequency and efficiency on marine bacteria than terrestrial or freshwater bacteria. Differences in prey susceptibility patterns on a panel of test bacteria separated the *Halobacteriovorax* isolates into three groups. The low nutrient medium (basal medium) yielded more predation positive results than the complex (peptone yeast extract) medium. This reveals the impact culture medium can have on susceptibility test results.

The prey susceptibility results reported by Taylor et al. (1974) have served as a resource for prey selection by other investigators and have been confirmed by most other studies. Variable results may occur under different experimental conditions. Nonetheless, susceptibility testing continues to be widely used to characterize and differentiate predator isolates in conjunction with molecular methods as comparative sequences of the 16S rDNA gene and whole-genome sequencing.

The superiority of *Vibrio* species as prey for marine *Bdellovibrio* (*Halobacteriovorax*) recovered from diverse sites is shown by results of an analysis by the authors of relevant reports in the literature (Table 1A). Miyamoto and Kuroda (1975) found that all of 12 *V. parahaemolyticus* strains isolated from different sources were susceptible to the predators. Ottaviani et al. (2018) reported similar results with all of 17 *Vibrio* strains, except the *V. alginolyticus* strains, being susceptible to a single *Halobacteriovorax* isolate recovered from the Central Adriatic Sea of Italy (Table 1A). None of the non-*vibrio* species (two strains of *E. coli*, *A. hydrophila, P. aeruginosa*) were susceptible. Enos et al. (2018) found that all *V. parahaemolyticus* isolates tested, a *Pseudomonas* soil isolate, and two strains of *E. coli* were susceptible to a *Halobacteriovorax* strain isolated from a Rhode Island (United States) estuary on a *V. parahaemolyticus* strain. The only species not susceptible was a freshwater *Acinetobacter* isolate.

**TABLE 1A:** A summary compiled from nine references of the most susceptible and non-susceptible bacteria to halophilic BALOs (*Halobacteriovorax*, *Bacteriovorax*, marine Bdellovibrios).

References	Environment	Primary prey^*a*^	Most susceptible bacteria^*b*^	Non-Susceptible bacteria
Marbach et al., 1976	Coast of Israel	*luminous strain LR-101*	*Vibrio* sp. (2) *Luminous* sp. MAV, *Aeromonas* sp. (2) *Pseudomonas* sp. S51, *Beneckea harveyi* 1	*Luminous* sp. W18*, *Beneckea harveyi* 126*, *Bacillus* sp. (G+), *Pseudomonas* sp. *L-1*
Sanchez-Amat and Torrella, 1989	Spanish Mediterranean Coastal Seawater and adjacent high salt ponds	Enriched natural bacteria population from sample sites	*V. parahaemolyticus*, *V. splendida*, *V. alginolyticus* (4)	Not listed
Sutton and Besant, 1994	Australian coastal waters	*V. alginolyticus*	*V. aestuarinus*, *V. alginolyticus*, *V. anguillarum, V. carchariae, V. campbellii, V. costicola, V. cholerae, V. diazotrophicus, V. fluvialis, V. furnissii, V. harveyi (3), V. hollisae, V. natriegens, V. ordalii, V. orientalis, V. pelagius (2), V. tubiashii, V. splendidus (2), Vibrio* sp. ACMM PM3, *V. vulnificus, V. pelagius, Alcaligenes aestus, Ps. bathycetes, Blastobacter* sp., *Photobacterium angustum, Achromobacter colinophorum, Ps. bathycetes, Escherichia. coli, Al. aquamarines, Al. aestus*	*Ps. atlantica* ACMM3, *Ps. aeruginosa, Ps. marina, Cytophaga marinoflava, Spirillum*-like sp., *V. gazogenes, V. mimicus*
Piñeiro et al., 2004	Great Salt Lake, UT, United States	*Not listed*	*V. cholera*	*V. vulnificus*
Cai et al., 2008	Shenzhen Bay, China	*V. parahaemolyticus*	*V. alginolyticus (9), V parahaemolyticus* (8), *V. fluvialis (7), V. cholerae* (5), *V. mimicus (4), V. anguillarum*,	*V, alginolyticus* strains (2), *V. cholera* 10–211, *V. fluvialis, V. parahaemolyticus* (2)
Richards et al., 2016	Delaware Bay sites, the Gulf Coast of Alabama	*V. parahaemolyticus*	*V. parahaemolyticus* (5)	*V. vulnificus* (2), *E. coli* (4), *V. alginolyticus, Salmonella enterica*
Kongrueng et al., 2017	Water and sediments in Thailand	*V. parahaemolyticus* (AHPND) cocktail of 4 isolates	*V. parahaemolyticus* (AHPND), *V. cholera*, *V. alginolyticus, V. vulnificus*, *V. parahaemolyticus* (clinical)	None of the bacteria tested
Enos et al., 2018	Rhode Island (United States) estuary, freshwater, soil	*V. parahaemolyticus*	*Vibrio* sp. (4), *E. coli* (2), *Pseudomonas* sp.	*Acinetobacter* sp.
Ottaviani et al., 2018	Central Adriatic Sea of Italy	*V. parahaemolyticus*	*V. parahaemolyticus* (7), *V. cholera* (6), *V. vulnificus* (2)	*V. alginolyticus* (2), *Sal. napoli, Sal.* Typhimurium, *E. coli* (2), *Ae. hydrophila* (2), *Ps. aeruginosa*

^*a*^Primary Prey means the prey bacteria used to isolate BALOs from the environment in the study. ^*b*^Numbers in the parentheses denote the number of strains tested in the study cited. *Resistant to 9 of 10 *Halobacteriovorax* isolates. G^+^, denotes Gram-positive prey.

**TABLE 1B:** A summary compiled from ten references of the most susceptible and non-susceptible bacteria to non-halotolerant BALOs from freshwater/terrestrial environments.

References	Environment	Primary Prey	Most susceptible prey	Non-Susceptible bacteria
Dias and Bhat, 1965	sewage and activated sludge		*Ps. fluorescens, E. coli, Sal. typhosa, Aerobacter aerogenes, Prot. morganii, Sal. paratyphi, Ps. aureofaciens, Ps. aeruginosa, Serratia marcescens*	*Al. faecalis, Bacillus megaterium* (G+), *Corynebacterium Barkeri* (G+), *Staphylococcus aureus* var. *citreus* (G+)
Klein and Casida, 1967	soil	*E. coli*	*E. coli* (27), *Aerobacter aerogenes, Erwinia atroseptica, Er. tracheiphila, Ser. kiliensis 187, Ser. marcescens 185*	*Agrobacterium radiobacter* 386, *Agrobacterium tumefaciens* 385, *Arthrobacter* sp. 8010, *Arthrobacter globiformis, Er. tracheiphila, Ritizobium japonicum 308a, Nocardia* sp. (G^+^)
Torrella et al., 1978	Various geographic locations in freshwater/soil		*Spirillum serpens, E. coli (2), Ps. putida ICBP 2484, Sal.* Typhimurium *LT-2, Proteus mirabilis, Aeromonas T1A, Ser. marcescens CDC 610265, Ae. hydrophila, ATCC 7966*	*Al. faecalis, Ae. hydrophyla UMOS-11, Achromobacter liquifaciens ATCC 17716*
Qian, 1994	Rivers in Chendu, China		*E. coli, F’s dysentery bacillus, Sal.* Typhimurium, *Ps. Aeruginosa, B. cereus* (G+)	*B. subtilis* (G+), *Saccharomyces* sp.
Jurkevitch et al., 2000	Soil, rhizosphere, root extract		Chromobacterium violaceum*, Enterobacter agglomerans*, Ps. Corrugate*, Ps. Syringae*, Er. carotovora subsp. carotovora 24**, Xanthomonas campestris**, E. coli**, *Agr. tumefaciens* IDI, Er. Amylovora**, Ps. putida***, Rhizobium cicer***	*Azospirillum brasilense, Er. carotovora* subsp. *carotovora* 2, *Agr. tumefaciens* C58****, Ser. Marcescens****, Ps. maltophilia****, Sinorhizobium meliloti (2)****, R. etli****, R. tropici****, *B. megaterium* (G^+^), *V. fluvialis*
Song, 2004	Rice Paddy water or rhizosphere, Korea	*Burkholderia glumae*	*Bur. glumae*	*Azo. brasilense*, *Bur. cepacia, Paenibacillus polymyxa*, *Pantoea herbicola, Ps. putida, Ps. syringe*, *Ser. marcescens*
Davidov et al., 2006b	Soil		*Agrobacterium* sp., *Agr. tumefaciens* C58, *Azo. brasilense* Cd, *E. coli* ML35, *Pectobacterium carotovorum*	Ps. corrugate, Ps. syringae*****
Dashiff et al., 2011	Laboratory strains	*E. coli*	*Acinetobacter* sp. (2), *Acin. baumanii (3) Acin. calcoaceticus, Acin. hemolyticus, Acin lwoffii (2), Aeromonas* sp. *(2), Bordetella bronchiseptica PIC 402, Bur. cepacia, Citrobacter freundii (3), Enterobacter aerogenes (4), Ent. amnigenus, Ent. cloacae (3), Ent. geriviae, E. coli (3), Klebsiella* sp. *(4), Listonella anguillarum, Morganella morganii (3), Prot. mirabilis (6) Prot. morganii, Prot. rettgeris, Prot. vulgaris (5), Ser. marcescens PIC 361, Ps. aeruginosa ATCC BAA-427, Ps. fluorescens PIC 105, Ps. syringae, Ps. putida, Sal. enterica, Ser. marcescens, Shigella flexneri, Shig. Sonnei, V. angulara, V. cholera, V. parahaemolyticus, Yersinia enterocolitica, Y. pseudotuberculosis*	*Campylobacter* sp. (2), *Ps. aeruginosa* (3), *Stenotrophomonas maltophilia. Enterococcus faecalis (G^+^)*, *Mycobacterium lacticola (G^+^) Mycobacterium smegmatis (G^+^) Sta. aureus (G^+^)*
Oyedara et al., 2016	Soil	*Klebsiella* sp. and *Salmonella* sp.	*Klebsiella* sp. (3), *Salmonella* sp. (4), *Ps. fluorescens, Ps. aeruginosa* (ATCC 27853), *Ps. putida*, *Pseudomonas* sp. DTB, *Enterobacter* sp., *Serratia* sp., *V. cholera*, *Alcaligenes* sp., *E. coli* (6), *Prot. mirabilis, Citrobacter freundii*	*E. coli* DH5α, *Stenotrophomonas* sp. (3), *Ps. syringae*, *Agr. tumefaciens, Rhizobium* sp., *Sta. aureus* (3) (G+), *Sta. epidermidis* (G+), *B. cereus* (G+), *B. thuringiensis* (G+)
Yu et al., 2017	Municipal waste sludge		*Klebsiella* sp., *E. coli, Raoultella* sp., *Enterobacter* sp*., Aeromonas* sp.	Not listed

*Susceptible to 4 of 5 BALO isolates. **Susceptible to 3 of 5 BALO isolates. ***Susceptible to 2 of 5 BALO isolates. ****Tested non-susceptible to 4 of 5 BALO isolates. *****Non-susceptible to 11 of 14 BALO isolates (other than original isolates). G^+^, denotes Gram-positive prey.

**TABLE 1C:** A summation from nine studies (see Table 1A) of bacteria species found most susceptible and non-susceptible to halophilic BALOs (*Halobacteriovorax*).

Most susceptible bacteria to halophilic BALOs	Frequency	NON-susceptible bacteria to halophilic BALOs	Frequency
*Vibrio* sp.	44	*E. coli*	6
*V. parahaemolyticus*	24	*V. alginolyticus*	5
*V. alginolyticus*	15	*V. vulnificus*	3
*V. cholerae*	14	*A. hydrophila, B. harveyi, V. parahaemolyticus, P. aeruginosa, V. fluvialis*	2
*V. fluvialis*	8	*Luminous* sp., *Ps. aeruginosa, Pseudomonas* sp., *Bacillus* sp., *Ps, atlantica, Ps. marina, Cytophaga marinoflava, Spirillum* sp., *V. gazogenes, V. mimicus, V. cholera, Sal enterica, Acinetobacter* sp., *Sal. napoli, Sal. Typhimurium*	1
*V. vulnificus*	4		
*V. mimicus*	4		
*V. harveyi*	3		
*Aeromonas* sp*., Pseudomonas* sp., *Ps. bathycetes, E. coli, Al. aestus, V. anguillarum, V. pelagius, V. splendidus*	2		
*Luminous* sp*., Beneckea harveyi, V. splendida, Photobacterium angustum, Achromobacter colinophorum, Al. aquamarines, V. aestuarinus, V. carchariae, V. diazotrophicus, V. campbellii, V. costicola. V. furnissii, V. hollisae, V. natriegens, V. ordali, V. orientalis, V. tubiashii, Blastobacter* sp.	1		

**TABLE 1D:** Summation of bacteria species found (from Table 1B) to be most susceptible and non-susceptible to non-halotolerant BALOs from freshwater/terrestrial environments.

Most susceptible bacteria to F/T BALOs	Frequency	NON-susceptible bacteria to F/T BALOs	Frequency
*E. coli*	43	*Sta. aureus*	5
*Prot. mirabilis*	8	*Stenotrophomonas* sp., *Ps. aeruginosa, Ps. syringe*	3
*Salmonella* sp.	7	*Campylobacter* sp., *Al. faecalis, B. megaterium, Agr. tumefaciens, Sinorhizobium meliloti*	2
*Klebsiella* sp.	6	*Agr. radiobacter, Agr. tumefaciens, Arthrobacter* sp., *Azo. brasilense, Ser. marcescens*	
*Ser. marcescens, Ps. aeruginosa, Prot. vulgaris*	5	*Er. tracheiphila, Ritizobium japonicum, Nocardia* sp., *Ae. hydrophyla, Achromobacter liquifaciens, B. subtilis, Saccharomyces* sp., *Er. carotovora, Bur. cepacia, Paenibacillus polymyxa, Pseudomonas putida, Ps. corrugate, Stenotrophomonas maltophilia, Enterococcus faecalis, Mycobacterium lacticola, Mycobacterium smegmatis, Sta. epidermidis, B. cereus, E. coli, Rhizobium* sp., *Pantoea herbicola, Corynebacterium barkeri. Ps. maltophilia, R. etli, R. tropici*	1
*Enterobacter aerogenes, Citrobacter freundii, Ps. putida*	4		
*Aeromonas* sp., *Acin. baumanii, Morganella morganii*	3		
*Enterobacter* sp., *P. fluorescens, Aerobacter aerogenes, Pseudomonas corrugata, Pseudomonas syringae, Prot. morganii, Bacillus* sp., *Acinetobacter* sp., *Acin lwoffii, Agr. Tumefaciens, Sal. Typhimurium*	2		
*V. cholera, V. parahaemolyticus, Sal. typhosa, Sal. paratyphi, Ps. aureofaciens, Er. atroseptica, Er. tracheiphila, Ser. kiliensis, Spirillum serpens, Ent. amnigenus, Ent. geriviae, Ent. Cloacae, A. hydrophila, Chromobacterium violaceum, Enterobacter agglomerans, Xanthomonas campestris, Er. carotovora, Burkholderia glumae, Er. amylovora, Azo. brasilense, Prot. rettgeris, Pectobacterium carotovorum, Bordetella bronchiseptica, Bur. cepacia, Acin. hemolyticus, Acin. calcoaceticus, Listonella anguillarum, Vibrio* sp., *Yersinia* sp., *Serratia* sp., *Alcaligenes* sp., *Shig. flexneri, Y. enterocolitica, Y. pseudotuberculosis, Raoultella* sp., *Shig. sonnei, V. angulara, Shigella* sp., *Rhizobium cicer*	1		

Unlike most studies, some investigators have used a primary prey other than *V*. *parahaemolyticus* to isolate *Halobacteriovorax* strains that were subsequently used to test the susceptibilities of selected bacteria strains. Marbach et al. (1976) used luminous bacteria strain LR-101, and Sutton and Besant (1994) used *V. alginolyticus* (Table 1A). These studies yielded results similar to predators isolated on *V. parahaemolyticus* in other investigations (Taylor et al., 1974; Schoeffield and Williams, 1990), and shows the diversity in predation capabilities of *Halobacteriovorax* isolated from different water bodies on the same bacteria species.

The susceptibility of seven bacteria species or strains isolated from Mediterranean coastal seawater and adjacent high salinity saltern ponds to 13 *Halobacteriovorax* strains isolated from the same sites were investigated (Table 1A) (Sanchez-Amat and Torrella, 1989). Interestingly, there was little difference between the susceptibility patterns of the bacteria isolated from seawater and those from the high salinity salt ponds with a single exception. This contrasts with the results of studies on *Halobacteriovorax* isolates from the Great Salt Lake (United States) (Piñeiro et al., 2004). The predation profile of six *Halobacteriovorax* isolates from the Great Salt Lake (GSL) (salinity range 40 and 170 ppt), was compared to isolates from waters in the Chesapeake Bay (Maryland, United States), Maryland coastal Atlantic Ocean, and the Virgin Islands (all with salinity < 35 ppt). Predator isolates were tested for predation on four unspecified bacteria isolated from the GSL, bacteria from the Mediterranean Sea, and *V. parahaemolyticus*, *V. cholera*, and *V. vulnificus*. The results revealed that the GSL predators preyed preferentially on the GSL bacteria in comparison to bacteria from the other locations. The difference was significant. This was one of the first reports that *Halobacteriovorax* prefers prey bacteria in their habitat over those from foreign habitats. The GSL predator isolates were all distinct from each other in prey susceptibility patterns. *V. parahaemolyticus* was preyed on by all *Halobacteriovorax* isolates, similar to results reported previously by Sanchez-Amat and Torrella (1989) from high saltwater ponds. However, *V. vulnificus* showed a much different profile, being resistant to all the GSL predator isolates, but susceptible to nearly all from ocean water.

Prior to 1990, most studies on the susceptibility of bacteria to *Halobacteriovorax* tested selected laboratory grown isolates of the predators (Taylor et al., 1974; Miyamoto and Kuroda, 1975; Marbach et al., 1976). Using a different approach, Sanchez-Amat and Torrella (1989) tested the susceptibilities of several different *Vibrio* and *Pseudomonas species* to the universe of *Halobacteriovorax* populations in samples of seawater and salt pond water in predator enrichment experiments. All of the tested prey species were susceptible to and supported the growth of some native *Halobacteriovorax* in the samples. The most efficient prey in three independent experiments was a *Vibrio* species (Table 2).

**TABLE 2:** *Bdellovibrio* (*Halobacteriovorax*) concentrations at 0 h and after 48 h in four independent auto enrichment experiments using coastal Mediterranean seawater.

		*Bdellovibrio* concn. (pfu/ml) in the enrichment flasks	
Expt	Host	0 h	48 h
A	*Vibrio alginolyticus* UM1	5	1.48 × 10^4^
	*Vibrio parahaemolyticus* UM1	4	3.48 × 10^4^
	*Pseudomonas* UM1	130	3.6 × 10^4^
B	*Vibrio alginolyticus* UM1	nd	2.8 × 10^4^
	*Pseudomonas* UM1	47	2.1 × 10^4^
C	*Vibrio alginolyticus* UM1	1	5.57 × 10^6^
	*Pseudomonas* UM1	4	2.7 × 10^6^
D	*Pseudomonas* UM1	0	2.56 × 10^6^

*From Sanchez-Amat and Torrella (1989). This shows the efficiency of the various bacteria in recovery of the *Halobacteriovorax*.*

**FIGURE 3:**
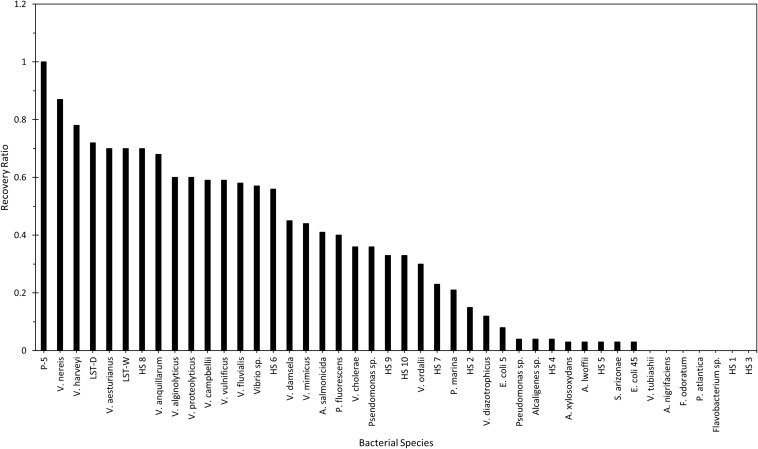
Quantitation efficiencies of various bacterial prey compared with that of *V. parahaemolyticus* P-5. Reprinted from Schoeffield and Williams (1990). Copyright (1990) American Society for Microbiology.

Using a similar approach, but different experimental design, Schoeffield and Williams (1990) evaluated 44 test bacterial species for their susceptibility and plating efficiency to the universe of cultivable *Halobacteriovorax* in water samples. Given the high efficiency of *V*. *parahaemolyticus* in the recovery of marine bdellovibrios (*Halobacteriovorax*) (Taylor et al., 1974; Sanchez-Amat and Torrella, 1989), it was used as the reference organism to which other species were compared. Water samples from a brackish tidal pond and an aquarium saltwater tank were plated for recovery of *Halobacteriovorax* using the double-agar overlay method with *V*. *parahaemolyticus* and each of the test bacteria. Two major findings were revealed. Firstly, a large number (74 to 84%) of the test bacteria were susceptible to *Halobacteriovorax* (Figure 3). Secondly, in every case, *V. parahaemolyticus*, the reference organism, was the most efficient at the recovery of *Halobacteriovorax* (a single exception in one trial of aquarium water could not be confirmed on repeat testing). Further, 97% of the *Halobacteriovorax* plaques that appeared on the test bacteria also produced plaques when transferred onto cultures of the *V. parahaemolyticus* reference strain. Collectively, other vibrios yielded significantly greater plaques, up to three times higher, than the non-*Vibrio* species. The most efficient bacteria in the recovery of *Halobacteriovorax* from aquarium water were many species of *Vibrio*, *E. coli*, and *Ps. fluorescens*. From the pond, the most efficient were *Vibrio* species. Less efficient were *P. fluorescens* and *E. coli*. Non-susceptible species from pond and aquarium waters were *Alteromonas nigrifaciens*, *Flavobacterium* sp., *V. tubiashii*, *V. ordalii*, *Acinetobacter lwoffii*, *Ps. atlantica*, and several other *Pseudomonas* species. The efficiency of other test bacteria in the recovery of *Halobacteriovorax* is shown in Figure 3. That the native *Halobacteriovorax* were capable of predation on a wide range of bacteria, including non-vibrio species, is an important factor in the sustainability of the predators in the environment as they require at least 5 logs ml^–1^ of prey, which typically is more than the abundance of most single species (Varon and Zeigler, 1978).

Few studies had addressed the susceptibility of autochthonous bacteria to native predators until the investigation by Rice et al. (1998). Autochthonous heterotrophic bacteria recovered from the water, sediment, and biofilm samples collected at various sites in the Chesapeake Bay system, and two brackish ponds, were tested for susceptibility to *Halobacteriovorax* predators isolated from the same site. *Pseudomonas* species, the dominant bacteria recovered, were highly susceptible (73 to 94%) to the *Halobacteriovorax* isolates. The susceptibility of *Vibrios*, the second-most isolated genus, was greater, ranging between 76 to 100%. The mean percent of susceptible bacteria from all type samples (water, sediment, surface biofilm) was 87.8% for *Vibrios* and 80% for *Pseudomonads*. The high susceptibility of *Pseudomonas* is surprising and contrasts with results from some other studies (Taylor et al., 1974; Sutton and Besant, 1994). There were greater numbers of *Pseudomonas* isolates (14) than *Vibrio* isolates (7) that were non-susceptible to the native *Halobacteriovorax*. This experimental approach appears to yield more accurate information on bacteria in the environment that can support the growth of the predators than results from experiments using selected laboratory isolates. Considering that the number of cultivable bacteria represents only a small fraction of the total bacteria present in environmental samples, several logs more prey bacteria were likely present in samples but were non-culturable. This is further evidence that the abundance of susceptible bacteria in coastal estuarine bodies is sufficient to support populations of *Halobacteriovorax*.

The results of the studies reviewed above show conclusively that *Halobacteriovorax* prey on many Gram-negative bacteria, and not all predator strains prey on the same bacteria. This suggests that the susceptibilities of bacteria inhabiting any environmental niche, and the predation efficiencies of the predators on them, may influence the composition and structure of the *Halobacteriovorax* community. However, practical methods to characterize BALO communities did not exist until after 1990 with the development of molecular phylogenetic methods that made possible detection and enumeration of distinct phylotypes of BALOs (Donze et al., 1991). This is demonstrated in the following two studies.

Over a tidal cycle in the Apalachicola Bay, Florida (United States), the following parameters were monitored, bacterial groups, *Bacteriovorax* (*Halobacteriovorax*) strains, salinity, and dissolved organic matter (DOM) (Chauhan et al., 2009). At low tide, higher concentrations of DOM were observed with a concomitant increase in bacterial and *Halobacteriovorax* numbers. Significantly greater numbers of γ-*Proteobacteria*, δ-*Proteobacteria*, *Bacteroidetes*, and high- G+C Gram-positive bacteria were seen. The more active predator population was *Bdellovibrio* phylogenetic clusters I and VI, typically associated with mesohaline regions. With increased salinity at high tides, the bacteria population shifted to a community dominated by α-*Proteobacteria*, β-*Proteobacteria*, and *Chlamydiales*-*Verrucomicrobia.* With changes in salinity and bacteria population came a shift in the predator community to one dominated by halophilic *Halobacteriovorax* clusters III, V, and X. The BALO species were significantly different (*p* < 0.001) than at lower tide. This is the first that the influences of these environmental factors on *Halobacteriovorax* communities have been demonstrated *in situ*.

Subsequently, Chen et al. (2011) investigated the responses of *Halobacteriovorax* phylotypes in an environmental water sample that was split into two subsamples one inoculated with *V. parahaemolyticus* and the other with *V. vulnificus*. The surprising results revealed that distinctly different *Halobacteriovorax* phylotype communities responded to the two *Vibrio* species. In an expanded experiment, eight bacteria species of marine and non-marine bacteria were used as prey. *Halobacteriovorax* Cluster IV was the predominant phylotype that grew on non-marine bacteria, whereas Cluster IX was predominant on marine prey (Chen et al., 2012). These results show a role for prey bacteria in shaping the community structure of *Halobacteriovorax* in aquatic environments. These findings are bolstered by supporting evidence from other studies (Richards et al., 2016).

Another example of differential predation is that of a non-halotolerant BALO isolate reported by Rogosky et al. (2006). When *B. bacteriovorus* 109J was added to a mixture of two prey cells, the predator preferentially preyed on one over the other. This suggests that selection of prey by *B. bacteriovorus* 109J is not a random occurrence. The components of the prey or predator cell that drives this specific selection remain unknown, however, a recent study offers some clues, including secondary metabolites (Mun et al., 2017).

The selective nature of predation on bacteria by *Halobacteriovorax* isolates was also reported by Richards et al. (2016). *Halobacteriovorax* isolates from Delaware Bay sites and the Gulf Coast of Alabama (salinities ∼ 30 ppt) recovered on *V*. *parahaemolyticus* preyed on other *V. parahaemolyticus* strains, but not on *V. vulnificus*, *V. alginolyticus*, *E. coli*, and *Salmonella enterica* (Table 1A). To the contrary, *Halobacteriovorax* isolated from a low salinity (∼0.5%) Delaware site, the Jones River, on *E. coli* and *Salmonella* species were capable of predation on all the *Vibrio* sp. and non-marine bacteria, demonstrating a broader prey range than the isolates from high salinity waters. These *Halobacteriovorax* from low salt waters were observed to grow at high salinities (5, 10, 20, and 30 ppt), but less efficiently than in lower salinities.

A summation of susceptible and non-susceptible bacteria to *Halobacteriovorax* from nine studies testing many different bacteria genera and species show *Vibrio* species as the dominant susceptible organism for the halophilic predators (Table 1A). Although other bacteria groups as the enteric bacteria, non-fermentative bacteria, and non-halophilic organisms are susceptible, none of these, individually or collectively, are so on the same scale as the *Vibrio* species. These results are consistent with the groundbreaking investigations on bacteria susceptible to *Halobacteriovorax* by Taylor et al. (1974), Schoeffield and Williams (1990) and others, and most recently confirmed by Najnine et al. (2020) in a study of the predators and potential prey in aquaculture systems. Our summation of the data from this study show among susceptible bacteria a predominance of *V. parahaemolyticus* and other vibrios. Collectively, these studies show vibrios to be closest among bacteria tested to being a universal prey for the *Halobacteriovorax.*

The summation of non-susceptible bacteria to *Halobacteriovorax* shown in Table 1C includes *E. coli*, some *Vibrio* species, *Ps. aeruginosa*, all of which are also listed as susceptible in Table 1C. Based on the analysis of the data from Table 1B, the most resistant was found to be E. coli followed by two Vibrio species. A comparison of the results of Table 1C shows that the susceptibility of bacteria to *Halobacteriovorax* and other BALOs may vary not only by species but also by different strains of the same species. It is known that the susceptibility of bacteria to *Halobacteriovorax* may be dependent on environmental factors, as Taylor et al. (1974) and Chen et al. (2018) have demonstrated with media of varying nutrient concentrations. It was found that high concentrations of nutrients may increase the resistance of bacteria to the predators.

Prey bacteria are not only the major nutrient source and microhabitat for *Halobacteriovorax* growth and multiplication, but also because the predators are dependent on prey for survival their occurrence in environmental niches will likely be determined by the abundance of prey. This has been shown to be the case in sediments and surface biofilm (Kelley et al., 1997). It has been shown by Chen et al. (2012) that prey bacteria also has the potential to select for specific subpopulations of *Halobacteriovorax* and shape the structure of predator communities. Considering the many ways in which prey are crucial to the survival, distribution, and structure of BALO communities, it is obvious that they are a critical determinant in the ecology of *Halobacteriovorax* in saltwater ecosystems.

#### Non-halotolerant BALOs and Prey

Non-halotolerant BALOs consist of several genera (*Bdellovibrio*, *Peredibacter*, *Bacteriovorax*) found in freshwater bodies, soils, sewage, animals, and plants. Generally, the bacteria that are susceptible to the freshwater and terrestrial (F/T) BALOs are Gram-negative and non-halophilic. A summary of susceptible and non-susceptible bacteria from 10 reports is shown in Table 1B. One of the sources in which F/T BALOs have been studied is sewage and sludge. In one such study, Dias and Bhat (1965) reported that *Pseudomonas fluorescence*, *Salmonella species*, *Aeromonas*, and *Proteus morganii* were the most efficient (Table 1B) bacteria for enumeration of the predatory bacteria from sewage and sludge. BALO plaques on *E*. *coli* were overgrown by bacteriophage plaques and were not countable. No BALOs were isolated on *Alcaligenes faecalis* NCIB 8158 and Gram-positive bacteria tested. In a more recent study on the enumeration of BALOs in municipal waste sludge, Yu et al. (2017) reported that five bacteria species, *Aeromonas*, *Klebsiella*, *Escherichia*, *Raoultella*, and *Enterobacter*, all supported BALO growth (Table 1B), but the *Klebsiella* species was slightly more efficient.

The wide use of *E*. *coli* for the isolation of F/T BALOs was perhaps influenced by reports such as Klein and Casida (1967) that all 25 *E. coli* serogroups tested were susceptible to two BALO strains isolated from soil on an *E*. *coli* prey (Table 1B). Less than 50% of other bacteria species from soil and other sources were susceptible. In a survey of over 20 reports published between 1963 and 1978 on environmental isolations of F/T BALOs, Varon and Shilo (1980) found that the most frequently used prey bacterium was *E. coli*, although it is not considered to be native to the environment, except sewage polluted areas.

Torrella et al. (1978) tested the susceptibility of 12 bacteria species representing nine genera to 12 non-halotolerant BALO laboratory strains isolated from different sources The results are summarized in Table 1B. An example of how susceptibility within the same species can vary is shown with *A. hydrophila* UOMS-11, which was not susceptible to any of the six predator strains tested, whereas *A.* hydrophila ATCC 7966 was susceptible to two of the 12 BALO strains. The authors note that although many of the bacteria tested were preyed upon, the size, morphology, and rate of growth of plaques sometimes differed between the prey bacteria. Another interesting observation was the effect of the incubation temperature on the susceptibility of one specie to one predator isolate. When incubated at 37°C prior to being tested for susceptibility, *Serratia marcescens* was preyed upon by *Bdellovibrio* SP1, but not so when first incubated at 30°C. This shows that the cultural conditions of the test bacterium can have an effect on its susceptibility. Investigating changes in the cell wall structure of *S*. *marcescens* when shifted from 30 to 37°C may yield some clues.

Many environmental investigations on F/T BALOs since the 1990s have involved isolates from soils and the rhizosphere. Jurkevitch et al. (2000) tested the susceptibilities of 22 bacteria species to five BALO isolates, three from local samples of soil, rhizosphere, and root extract, respectively, one from tomato plant roots, and *Bdellovibrio* (Bd) 109J (Table 1B). Surprisingly, most bacteria, 10 of the 22, were susceptible to Bd. 109J, the only nonnative predator strain. In a subsequent, more comprehensive study, BALOs in soil samples were quantitatively recovered on six different bacteria (Davidov et al., 2006a). *Ps. syringae* yielded nearly five times greater numbers of BALO plaques than the second most efficient prey, *E. coli* ML35 (Table 3).

**TABLE 3:** Quantification of plaque–forming units (PFUs) by BALOs retrieved from an En–HaNaziv soil^*a*^.

Substrate organism	10^2^ PFU per gram of soil (dry weight)^*b*^
*Agrobacterium tumefaciens* C58	2.26 ± 0.28
*Azospirillum brasilense* Cd	0.76 ± 0.12
*Escherichia coli* ML35	5.08 ± 0.6
*Pectobacterium carotovorum* ssp. *carotovorum*	3.80 ± 0.28
*Pseudomonas corrugata*	3.12 ± 0.4
*Pseudomonas syringae*	24.60 ± 2.1

*Reprinted with permission from Davidov et al. (2006a). The efficiency of the recovery of BALOs by the various substrate bacteria is shown. ^*a*^Determined on double layered HEPES agar. Total bacterial count on nutrient agar was 2.7 × 10^7^ CFU per gram of soil. ^*b*^Representative results of two experiments.*

Davidov and Jurkevitch (2004) evaluated 71 BALO isolates from different geographical locations and sources. The most frequently used bacteria for BALO isolations were *E. coli*, *Ps. corrugata*, and *Erwinia.* Of these, *E*. *coli* yielded more BALO isolations.

Two strains of BALOs isolated from soils in Mexico (Oyedara et al., 2016) on *Klebsiella* sp., and *Salmonella* were tested for predation on 36 bacterial strains. The first predator strain preyed on 13 of the bacterial strains and the other on 22. Of the four *Salmonella* strains, all proved susceptible to both BALO strains (Table 1B). Among seven *E. coli* strains, six were susceptible to one BALO strain and four to the other. *Agrobacterium tumefaciens* (CDBB-B-1042) and *Ps. syringae* were resistant to both BALO strains, whereas, in previous studies, both were susceptible (Jurkevitch et al., 2000). Several Gram-positive bacteria species tested were resistant (Table 1B).

Few studies on BALOs in freshwater bodies have been reported in the past two decades. In a recent report, Sar et al. (2015) described the isolation of 53 BALOs from freshwater systems in Nigeria using three bacteria species. The frequency of isolation of BALOs on the three prey bacteria was 79.2% on *E. coli*, 18.86% on *S. typhi*, and 1.88% on *Shigella* spp.

The susceptibility of nearly 80 human pathogens and multidrug-resistant bacteria to *B. bacteriovorus* 109J, originally isolated from soil, was reported by Dashiff et al. (2011) (Table 1B). *Proteus* species *were the most susceptible to the species tested* (Table 1B). *Proteus vulgaris* PIC 365 was the most efficient prey showing an 8-log reduction at 48h incubation. This was followed by two *E. coli* strains with a 7-log reduction. Not susceptible were *Campylobacter*, *Enterococcus faecalis*, and *Mycobacterium*.

The investigators also tested Bd 109J predation on different combinations of two genera of bacteria in mixed culture. As a control, BALO was co-cultured with each genus singularly. The results revealed the predator had reduced the counts of the mixed prey comparable to that observed in single prey control cultures. The study did not show if the predator’s rate of predation on the two different prey varied during the incubation period, as was reported previously by Rogosky et al. (2006).

Dashiff and Kadouri (2011) tested the susceptibility of bacteria associated with periodontitis to *Bd.* 109J. All 10 serotypes of *A*. *actinomycetemcomitans* tested were susceptible, as was *E*. *corrodens* and *F. nucleatum* 1594. *Prevotella intermedia*, *Porphyromonas gingivalis*, and *Tannerella forsythia* were not susceptible. All of these are bacteria that *Bd.* 109J from soil may not have encountered previously. This shows the broad prey range of *Bd.* 109J.

From the data in Table 1B, a sum of the various susceptible and non-susceptible bacterial species to F/T BALOs across the 10 studies were calculated. The results are shown in Table 1D. *E. coli* was by far the most dominant susceptible species. The next most susceptible group was various other species of enteric bacteria. How other susceptible species ranked is shown in Table 1D. The predominance of *E. coli* is likely a function of the fact that it is also the most frequently used bacterium for the recovery of BALOs and is most often incorporated in susceptibility testing. The species used to recover BALOs which are then used to test the susceptibility of other strains of the same species, are likely to be susceptible. Nevertheless, the ranking of *E. coli* as the most susceptible is consistent with results from other studies (Klein and Casida, 1967; Varon and Shilo, 1980). An exception is a report by Najnine et al. (2020) that examined BALOs for use in aquaculture and their prey. *A. hydrophila* was the most susceptible species. *E. coli* is also used more than other non-halophilic bacteria in susceptibility tests of bacteria for the saltwater *Halobacteriovorax*.

Many Gram-negative bacteria have been found to be susceptible to BALOs. The results of numerous studies show *E*. *coli* to be the most frequently used and often most efficient bacteria for recovery of F/T BALOs. However, few of the bacteria used to recover F/T BALOs from environmental samples are as efficient (usually below 60%) as the vibrios in the recovery of the halophilic *Halobacteriovorax* predators (typically 80% or above). In both cases, non-fermentative species tend to be more resistant. The reasons for bacteria resistant to BALOs remain a mystery. Some clues may be found in secondary metabolites such as indole (Dwidar et al., 2015) and cyanide (Mun et al., 2017) produced by some bacteria. Both appear to slow or inhibit the motility of BALOs, which would prevent predation. The search for other such substances and analyses of cell wall structures are important areas to pursue to learn more about BALO resistant mechanisms in bacteria.

## The Impact of Temperature

Temperature is another major environmental factor impacting the survival and ecology of the BALOs. The characterization of new isolates has typically included the predator’s temperature growth range and optima. The collective results from many studies confirm that the non-halotolerant F/T BALOs grow better at a higher temperature range, between 30 and 37°C, than the saltwater *Halobacteriovorax*, that grow optimally between 20 and 30°C, although some strains grow well at 35°C. *Bdellovibrio*-like bacteria have been isolated from hot springs. The mean water temperature at the sampling site were ~91°C ±3 and ~57°C ±2 for the water and mat surfaces, respectively. Unlike typical BALOs, these were epibiotic predators that did not penetrate the outer cell membrane of its prey, but attached side on with the prey (Sangwan et al., 2015).

The format of most temperature studies has been either laboratory experiments on pure cultures of BALO strains or environmental field investigations in which correlations between BALO numbers and temperatures were examined.

Miyamoto and Kuroda (1975) investigated the range and effects of temperature on predation of a *Halobacteriovorax* strain isolated on *V. parahaemolyticus* in the winter from Osaka Bay in Japan (Table 4A). In one experiment, the number and growth of *Halobacteriovorax* plaques observed on the prey, *V. parahaemolyticus*, were similar at room temperature (ranging from 4 to 15°C within a day), 20, 25, and 30°C. However, no plaques were observed at 5 and 35°C. When these plates were then moved to 25°C, plaques appeared on the original 5°C plate, but not on the original 35°C plate. In another experiment, co-cultures of *Halobacteriovorax* and prey in seawater were incubated at various temperatures. The optimum growth was observed between 25 and 30°C. At 35°C, growth was inhibited, and the numbers of the predators in the initial inoculum had declined to one-half in 1 day and one-tenth by 3 days. In a subsequent experiment, a suspension of *Halobacteriovorax* was exposed for 30 min to temperatures of 40, 45, and 50°C and then plated. Approximately one-half of the original number survived the 40°C treatment and formed plaques when plated with prey, but none exposed to the higher temperatures produced plaques. The results show that this *Halobacteriovorax* isolate did not survive well or at all, at temperatures of 40°C or above. These results have been confirmed by other investigators.

**TABLE 4A:** A summary compiled from references of the field studies on the role of temperature on BALOs.

References^*a, b*^	Environment	Primary prey	Temperature Range (°C)	Correlation cofficient r value (probability value) of number of BALOs and Temperature
Fry and Staples, 1976^*a*^	(1) River Water; (2) Sewage	*E. coli* or *Achromobacter* sp.	Not given	(1) 0.115 (NS*); (2) 0.400 (*p* < 0.001)
Williams et al., 1982^*b*^	Patuxent River, MD, United States (water, 3 sites)	*V. parahaemolyticus*	5.6–26	0.24–0.41 (*p* < 0.05)
Williams, 1988^*b*^	Patuxent River, MD, United States (sediment, 3 sites)	*V. parahaemolyticus*	5.4–26.5	0.3022 (*p* = 0.0001)
Sutton and Besant, 1994^*b*^	Great Barrier Reef region (3 sites), Australia	*V. alginolyticus*	23–29	0.34–0.60 (*p* < 0.001)
Richards et al., 2013^*b*^	(1) Delaware Bay**; (2) Gulf of Mexico; (3) Hawaii	*V. parahaemolyticus*	(1) 5–27; (2) 12.2–31; (3) 24–25	(1) 0.65*** (*p* ≤ 0.0001); (2) −0.585 (*p* ≤ 0.0001); (3) Not provided (*p* > 0.05)
Ottaviani et al., 2018^*b*^	Mussel farm area in Adriatic Sea, Italy	*V. parahaemolyticus*	9–22	0.96

^*a*^Studies on Freshwater/terrestrial BALOs. ^*b*^Studies on Halophilic BALOs. *NS, not significant. **Four sites. ***from site 4; no correlation at other sites.

**TABLE 4B:** A summary compiled from references of the laboratory studies on the role of temperature on BALOs.

References^*a, b*^	Source of Strains	Primary prey	Temperature Growth Range (°C)	Optimal Temperature (°C)
Varon and Shilo, 1968^*b*^	Other labs	*E. coli*	15–40	30–35
Seidler and Starr, 1969a^*b*^	Depositories	*E. coli*	25–38*	30–35
Miyamoto and Kuroda, 1975^*a*^	Osaka Bay, Japan	*V. parahaemolyticus*	Room temp (4–15) −30	20–25
Marbach et al., 1976^*a*^	Mediterranean Coast of Israel	Luminous strain LR-101	15–35**	25
Filip et al., 1991^*b*^	Sewage plant, Langen, Germany	*E. coli*	18–30	26–30
Jackson and Whiting, 1992^*b*^	Lab strains	*E. coli*	15–30	30
Fratamico and Cooke, 1996^*b*^	Depositories and Other Labs	*E. coli*	12–37	30–37
Chen et al., 2018^*a*^	Gulf of Mexico, United States		10–37	*25–37*

^*a*^Studies on Halophilic BALOs. ^*b*^Studies on Freshwater/terrestrial BALOs. *Lowest temperature tested was 25°C. **1 of 10 strains grew at 10°C.

Marbach et al. (1976) found that the optimum temperature range among 10 *Halobacteriovorax* isolates was between 15 and 35°C (Table 4B), and the time of the earliest plaque formation and best plating efficiency occurred at 25°C. In contrast to the report by Miyamoto and Kuroda (1975), nearly all strains were capable of forming plaques at 35°C and one at 40°C. Exceptions were with a strain that grew between 15 to 30°C and another strain that grew at a low of 10°C. In a 2004 report (Baer et al., 2004), the temperature growth range described for F/T BALO strains was 15–35°C (with a few exceptions), and for halophilic strains AQ and SJ, 15–30°C, and for strain JS 15–35°C. These results show variability among some strains of both F/T BALOs and *Halobacteriovorax*. The basis for this variability could be due to the temperature in the environment from which the predators were recovered and have adapted.

In what may be the first report describing a seasonal distribution for halophilic BALOs (*Halobacteriovorax*), Williams et al. (1982) recovered significantly greater numbers of *Halobacteriovorax* PFUs from the water column of an estuary during the warmer months than in winter (January, February, March). Additionally, a positive correlation was found between the numbers of predators and temperature (Table 4A). In some cases, in the winter months, *Halobacteriovorax* was not recovered. How, and where, the predators survived at the lowest temperatures were a mystery. Sediment was a consideration. This was addressed in a follow-up study of *Halobacteriovorax* in estuarine sediments over an annual cycle (Williams, 1988). *Halobacteriovorax* numbers correlated positively with temperature changes (Table 4A). At low temperatures in the winter, small numbers of the predators were recovered from sediments even when not detected in the water column. As the temperature increased in spring and summer months, the numbers of predators in sediment were observed to increase first, followed later by increases in the water column. Based on the results, it is apparent that *Halobacteriovorax* could better survive the winter in sediments than in the water column, and that sediments play a major role in their seasonal cycle, growth, and overall ecology.

Another major ecosystem for *Halobacteriovorax* communities and ecology is the epibiota or biofilm on surfaces in aquatic systems. Williams et al. (1995) reported that the predators were most frequently and consistently recovered from biofilm scraped from oyster shell surfaces compared to water, sediments, zooplankton, and plants, and was the only sample material with a 100% recovery rate. Recovery from water and sediment samples was 79 and 44%, respectively. At all temperatures, the abundance of *Halobacteriovorax* recovered from biofilm was also significantly greater than the numbers from the other samples. Below 10°C, the number and frequency of recovery of the predators from all samples were reduced, except for surface biofilm. In a first *in situ* study on the association of *Halobacteriovorax* with surfaces (Williams et al., 1995), the temperature was found to be a major factor.

Several studies covering a wide geographical range have reported seasonal cycles of *Halobacteriovorax*. The numbers of *Halobacteriovorax* in three Australian tropical marine habitats were found to be statistically correlated to seasonal seawater temperature (Sutton and Besant, 1994). Welsh et al. (2015) investigated *Halobacteriovorax* in corals at Pickles Reef (25° 00′ 05″ N, 80° 24′ 55″ W) within the Florida Keys Reef Tract, United States that preyed on putative coral pathogens, *Vibrio corallyticus* and *Vibrio harveyi*, and found that predator-prey interactions were affected by changing thermal regimes. Analysis by co-occurrence networks showed that interactions of the predators with other bacteria were under the influence of temperature.

A seasonal study of *Bacteriovorax* (*Halobacteriovorax*) by Richards et al. (2013) at six geographically dispersed locations included four sites in the Delaware Bay, the Gulf Coast of Alabama, and coastal waters in Hawaii (Table 4A). In Delaware Bay, predators were recovered from the four sites monthly, except February 2013, when the water temperature was lowest. No correlation was found between temperature and *Halobacteriovorax* numbers at the sites except for Site 4, a riverine site, and the most inland. There *Halobacteriovorax* counts were significantly higher (*p* ≤ 0.0001) in summer than winter. The opposite was observed in the Gulf Coast of Alabama with significantly more *Halobacteriovorax* (*p* ≤ 0.0001) during the winter, resulting in a negative correlation between temperature and *Halobacteriovorax* counts (Table 4A). This contrasts with results from most seasonal studies on *Halobacteriovorax* in temperate zones. Further investigation is needed to determine if this was an anomaly or is a repeated pattern. In Hawaiian waters, as expected, no significant seasonal differences (*p* > 0.05) in *Halobacteriovorax* counts were found where little variation in water temperature was observed.

In the Adriatic Sea (Ottaviani et al., 2018), a monthly survey of *Halobacteriovorax* counts over an annual cycle showed a strong positive correlation between *Halobacteriovorax* counts and water temperature (Table 4A). In Taiwan, Pan et al. (1997) reported the seasonal occurrence of *Halobacteriovorax* in coastal waters and aquaculture ponds.

Following a dearth of studies on BALOs in freshwater bodies over the past three decades, a few recent reports have emerged. Paix et al. (2019) reported seasonal effects on the numbers of F/T BALOs in Alpine Lakes influenced primarily by temperature and depth, particularly, for *Peredibacter starrii*, the most abundant of the F/T BALO families detected in the lakes. Ezzedine et al. (2020) found in Lake Geneva in France, seasonal fluctuations in numbers of *Bacteriovoracaceae*, *Bdellovibrionaceae*, and *Predibacteracea*e related to temperature. The seasonal occurrence of BALOs shows their population to be actively and continuously changing, but with stability, that seems to repeat itself yearly.

The survey of studies shows a rather distinct difference in the temperature growth range and optima between the saltwater *Halobacteriovorax* which typically grows at a lower temperature (20 to 30°C) and the F/T BALOs, which prefer a higher temperature (30 to 35°). There are few exceptions with both groups. In the environment, low temperatures at 15°C or below appear to have the greatest impact on the predators reducing their numbers significantly and contributing to the seasonality of the predators. With warming temperatures, the BALO numbers increase. An interesting observation is that a seasonal effect is observed with *Halobacteriovorax* even in locations where the temperature range is relatively small (23 to 29°C) such that the low temperature is relatively high as compared to the temperature lows in temperate climates. This was observed by Sutton and Besant (1994) (Table 4A).

## Conclusion

This review covers nearly 60 years of laboratory and field investigations on the impacts of salinity, prey, and temperature on both halophilic *Halobacteriovorax* and non-halotolerant BALOs. The results show definitively that environmental factors regulate the geographical and seasonal distribution of BALOs. Sodium chloride and other cationic salts appear to be the greatest factor. Salinity divides the BALOs into two major groups, the family *Halobacteriovoracaceae* that require salts for growth, and the families *Bdellovibrionaceae* and *Bacteriovoracaceae* that are inhibited by even low concentrations of NaCl. The *Halobacteriovorax* are further differentiated based on which of the three salinity zones (polyhaline, mesohaline, and oligohaline) they are typically found. In addition to sodium chloride, *Halobacteriovorax* are dependent on cations that play vital roles in BALO cellular functions. A deficiency of KCl in growth medium impairs motility, a lack of Mg^2+^ causes loss in cell wall integrity, and Ca^2+^ insufficiency results in inhibition of continuous predation and multiplication cycles. Temperature growth range and prey preferences also distinguish the *Halobacteriovorax* and F/T BALOs. The *Halobacteriovorax* grow more efficiently at lower temperatures (≤30°C) and the F/T BALOs at higher temperatures (≥30°C). In the case of *Halobacteriovorax*, a true seasonal distribution in temperate climate zones is established. As to prey preferences, the more efficient prey for the *Halobacteriovorax* are *Vibrio* sp. and other marine bacteria, whereas prey for the F/T BALOs are generally non-marine bacteria, the most widely used being *E*. *coli*. For both groups, *E*. *coli* is relatively efficient. Although the role of prey in selecting the population of BALOs in any niche is now evident, the predators simultaneously exert some control on the population of its prey, and in the process, bring structural changes to the bacterial community. This is likely a dynamic process with continuous alterations within the bacterial community caused by the predators, the extent of which may be dependent on temperature and salinity. Many unknown questions remain regarding the predator mechanisms for prey recognition, salinity and temperature adaptation, and re-structuring of bacterial communities. These require further investigation, as summarized below. In this review, we have shown that environmental factors do regulate the existence and ecology of BALOs in a major way and described the impacts of this regulation on this unique group of predatory bacteria.

## Directions for Future Study

Although this review serves to summarize into a single treatise the results of many studies on the ecology, behavior, and survival of BALOs, it barely penetrates the surface of the depth of information yet to be uncovered. Many questions have not been thoroughly investigated, and much remains to be done to address them. We would not want to complete this review without suggesting some new directions for future study. Still unknown are the mechanisms by which BALOs are able to differentiate between prey and non-prey bacteria. What are the factors that drive the *Halobacteriovorax* or saltwater BALOs to generally prey more efficiently on *Vibrio* species than on non-*Vibrio* species, and particularly many *Pseudomonas* species and other non-fermentative Gram-negative bacteria? Unlike many non-marine species, what makes *E*. *coli* a relatively efficient prey for both halophilic and non-halotolerant BALOs? Is the mechanism by which this occurs controlled within the BALO cell or the prey? Why do BALOs not prey intraperiplasmically on Gram-positive bacteria? Following up on a few reports included in this review, do BALOs kill Gram-positive bacteria and derive some nutritional benefits from the interaction? If so, do all BALOs have this capability, and under what conditions? Are all Gram-positive bacteria then vulnerable to BALOs? Currently, there is a lack of uniformity in methodologies across different laboratories in testing bacteria for susceptibility to BALOs, which remains a tool that yields valuable information for characterizing the predators. This makes variable results from laboratories using different protocols difficult to interpret. How to bring about accepted universal protocols for prey susceptibility studies that are widely accepted and as is the case for testing antibiotic sensitivity? How do BALOs survive in low prey environments as in open oceans or other oligotrophic environs? There is a need for more environmental and ecological studies on BALOs in freshwater systems, as most studies have been in saltwater bodies. Another area in which there is little information is the properties of BALO phylotypes. Do isolates within the same cluster have the same or very similar characteristics? Do BALOs of each cluster correlate with other environmental factors other than salinity, such as temperature and prey? To pursue this line of research, the use of wild-type predatory BALOs along with their derivative prey-independent mutants should be helpful in characterizing phylotypes as the BALOs are not easily grown from nature in pure culture.

To improve our understanding of BALOs in nature, it is critical to focus on them in bacterial community profiling surveys, both as components of the community and as a factor driving β-diversity. An important step in this aspect is recognizing BALO-specific key genes, preferably related to predation. Metagenomics and pan-genome surveys will be helpful in the identification of such genes beneficial for understanding BALO presence and contributions in different ecosystems. Investigations on population shifts targeting BALO-specific targeted gene(s) along environmental gradients will help advance knowledge on how environmental factors as temperature, salinity, nutrients, and protist grazing or viral lysis affect predator communities across space and time. Flow cytometry can be helpful to probe BALO specific genes and sort intact cells for in-depth investigation of prey-predator relationships. Another approach to explore predator-prey interactions, and the effects of environmental factors on coexistence within the community is co-occurrence network analysis. This will enable exploring BALOs in a variety of natural settings.

Grossly understudied are the interactions between BALOs and other bacterial predators such as bacteriophages and protists. Only a few studies have been reported (Chen and Williams, 2012; Johnke et al., 2017; Hobley et al., 2020). The issue of intraguild predation has been recently discussed by Kuppardt-Kirmse and Chatzinotas (2020). Related to this is the need for a greater understanding of the role of BALO predation and lyses of its’ prey in the cycling of nutrients through the microbial loop. Logic calls for these areas to be investigated to gain a better understanding of the field of biogeochemistry. Hopefully, this review will serve as a platform for new investigations in the areas identified above and others.

## Author Contributions

HW conceived the original idea. HW and HC co-wrote the manuscript, contributed to revision, and approved the submitted version. Both authors contributed to the article and approved the submitted version.

## Conflict of Interest

The authors declare that the research was conducted in the absence of any commercial or financial relationships that could be construed as a potential conflict of interest.
