# Supplementary material for: Environmental Regulation of the Distribution and Ecology of Bdellovibrio and Like Organisms
Source: Front Microbiol. 2020 Oct 29;11:545070. doi: 10.3389/fmicb.2020.545070 (PMC7658600; doi:10.3389/fmicb.2020.545070)
Supplement: Supplementary file 1 [file Table_1.docx]

Table S1. Host activity spectra of non-halotolerant *Bdellovibrio* strains isolated from soil samples (modified from Table 2 in Stolp and Starr, 1963)

|  | 1 | 2 | 3 | 4 | 5 |
| --- | --- | --- | --- | --- | --- |
|  | *Bd.* 128 | *Bd.* 321 | *Bd.* A3.12 | *Bd.* 100  *Bd.* 109  *Bd.* 110  *Bd.* 118  *Bd.* 120  *Bd.* 127  *Bd.* 233 | *Bd.* 101  *Bd.* 114 |
| *Erwinia amylovora* EA137 | - | - | - | + | + |
| *Erwinia* carotovora EC153 | - | - | - | + | + |
| *Agrobacterium* *tumefaciens* C113A | - | - | - | - | - |
| *Agrobacterium* *radiobacter* TR1 | - | - | - | - | - |
| *Escherichia* *coli* B2262 | - | - | - | + | + |
| *Aerobacter* *aerogenes* 2001 | - | - | - | + | + |
| *Pseudomonas* *polycolor* PP2 | - | - | - | - | - |
| *Acetobacter* *aceti* 2108 | - | - | - | - | - |
| *Proteus* *mirabilis* | - | - | - | - | + |
| *Caulobacter* sp. CB15 | - | - | - | - | - |
| *Pseudomonas* *aeruginosa* 2019 | - | - | - | - | - |
| *Serratia* *marcescens* 2031 | - | - | - | + | + |
| *Bacterium* *stewartii* SS12 | - | - | - | + | + |
| *Aeromonas* sp. 2326 | - | - | - | + | + |
| *Rhizobium* *leguminosarum* 2067 | - | - | - | - | - |
| *Protaminobacter* *rubrum* 2144 | - | - | - | - | - |
| *Pseudomonas* *solanacearum* PS138 | - | - | + | + | + |
| *Aerobacter* *cloacae* 2112 | + | - | - | - | - |
| *Pseudomonas* *caryophylli* PC102 | - | - | + | - | + |
| *Rhodospirillum* *rubrum* S1 | - | - | - | + | + |
| *Pseudomonas* *phaseolicola* ATCC 11355 | - | + | + | + | + |
| *Pseudomonas* *tabaci* NRRL B877 | - | + | + | + | + |
| *Pseudomonas* *fluorescens* ATCC 12633 | - | + | + | + | + |
