# Supplementary material for: Environmental Regulation of the Distribution and Ecology of Bdellovibrio and Like Organisms
Source: Front Microbiol. 2020 Oct 29;11:545070. doi: 10.3389/fmicb.2020.545070 (PMC7658600; doi:10.3389/fmicb.2020.545070)
Supplement: Supplementary file 2 [file Table_2.docx]

Table S2. Susceptibility pattern of various marine and non-bacteria species to 13 isolates of Marine Bdellovibrios (*Halobacteriovorax*) recovered from coastal waters of Oahu, HI USA. The original table listed susceptibility results observed on two media, yeast extract agar, and basal medium agar. We have modified the original figure by combining the results from the two media by recording a positive result if plaques occurred on either of the two media and a negative result if on both media no plaques were observed. Modified from Taylor et al (1974).

|  | **Bd-1** | **Bd-2** | **Bd-3** | **Bd-4** | **Bd-5** | **Bd-6** | **Bd-7** | **Bd-8** | **Bd-9** | **Bd-10** | **Bd-11** | **Bd-12** | **Bd-13** |
| --- | --- | --- | --- | --- | --- | --- | --- | --- | --- | --- | --- | --- | --- |
| **Facultatively anaerobic marine eubacteria** | |  |  |  |  |  |  |  |  |  |  |  |  |
| *Beneckea campbellii* 40 | + | + | + | + | + | + | + | + | + | + | + | + | + |
| *B. neptuna* 74 | + | + | + | + | + | + | + | + | - | + | + | - | + |
| *B. nereida* 80 | + | + | + | + | + | + | + | + | + | + | - | + | + |
| *B. pelagia* 99 | + | + | + | + | + | + | + | + | - | + | - | + | + |
| *B. natriegens* 107 | + | + | + | + | + | + | + | + | + | + | + | + | + |
| *B. parahaemolytica* 113 | + | + | + | + | + | + | + | + | - | + | + | + | + |
| *B. alginolytica* 118 | + | + | + | + | + | + | + | + | + | + | - | + | + |
| *B. nigrapulchrituda* 164 | - | - | - | - | - | - | - | - | - | - | - | - | - |
| *B. harveyi* 384 | + | + | + | + | - | - | - | - | - | + | + | + | + |
| *Photbacteriu fischeri* 61 | + | + | + | + | + | + | + | + | - | + | + | - | - |
| *P. phosphoreum* 447 | + | + | + | + | + | + | + | - | - | + | + | + | + |
| *P. mandapamensis* 480 | + | + | + | + | + | + | + | + | - | + | + | + | + |
| Group B-2 68 | + | + | + | + | + | + | + | + | + | + | + | + | + |
| Group C-2 324 | + | + | + | + | - | + | - | - | - | - | + | - | + |
| Group E-2 94 | + | + | + | + | - | + | + | - | + | + | - | + | + |
|  |  |  |  |  |  |  |  |  |  |  |  |  |  |
| **Non fermentative marine eubacteria** | |  |  |  |  |  |  |  |  |  |  |  |  |
| *Alteromonas communis* 8 | + | + | + | + | - | - | - | - | - | + | + | + | + |
| *A. vaga* 40 | - | - | - | - | - | - | - | - | + | + | + | - | - |
| *A. macleodii* 107 | - | - | - | - | - | - | - | - | - | - | - | - | - |
| *A. haloplanktis* 121 | - | + | + | + | + | - | - | - | - | - | - | - | - |
| *Pseudomonas doudoroffii* 70 | + | + | + | + | + | + | + | + | + | - | - | + | - |
| *P. marina* 140 | - | - | - | - | - | + | + | + | + | - | - | + | - |
| *P. nautica* 179 | + | + | + | + | - | + | + | + | - | + | + | + | + |
| *Alcaligenes pacificus* 62 | - | - | - | - | - | - | - | - | - | - | - | - | - |
| *A. cupidus* 79 | - | - | - | - | - | + | + | + | + | + | + | + | + |
| *A. venustus* 86 | - | - | - | - | - | - | - | - | - | - | - | - | - |
| *A. aestus* 134 | - | - | - | - | - | - | - | - | + | + | + | + | - |
| Group B-1 51 | - | - | - | - | - | + | + | + | + | + | + | + | + |
| Group B-2 54 | - | - | - | - | - | + | + | + | + | + | - | + | + |
| Group G-1 146 | + | - | + | - | - | + | - | - | + | + | + | + | + |
|  |  |  |  |  |  |  |  |  |  |  |  |  |  |
| **Non-marine eubacteria** |  |  |  |  |  |  |  |  |  |  |  |  |  |
| *Escherichia coli* B ATCC11303 | - | - | - | - | - | + | + | - | + | + | + | + | + |
| *Salmonella typhimurium* suc LL | - | - | - | - | - | + | + | + | + | + | + | + | + |
| *Aerobacter aerogenes* ATCC 13048 | - | - | - | - | - | + | + | + | + | - | + | + | - |
| *Aeromonas formicans* ATCC 13137 | + | + | + | + | + | + | + | + | + | + | + | + | + |
| *Vibrio cholerae* ATCC 14035 | + | + | + | - | + | + | + | - | - | + | + | + | + |
| *Acinetobacter calco-aceticus* 46 | - | - | - | - | - | - | - | - | - | + | - | - | - |
| *Pseudomonas aeruginosa* 45 | - | - | - | - | - | - | - | - | - | - | - | - | - |
| *P. fluorescens* 192 | - | - | - | - | - | - | - | - | - | + | + | - | - |
| *P. putia* 90 | + | + | - | - | - | - | - | - | - | + | + | - | - |
| *P. acidovorans* 14 | - | + | + | - | + | + | + | + | + | + | - | - | - |
| *P. testosteroni* 138 | - | - | - | - | - | + | + | + | + | + | + | + | + |
| *P. pseudoalcaligenes* 63 | - | - | - | - | - | - | - | - | - | + | + | - | - |
| *P. stutzeri* 223 | + | + | + | + | + | + | + | + | + | + | + | - | - |
